# Supplementary material for: Computational Investigation of 1, 3, 4 Oxadiazole Derivatives as Lead Inhibitors of VEGFR 2 in Comparison with EGFR: Density Functional Theory, Molecular Docking and Molecular Dynamics Simulation Studies
Source: Biomolecules. 2022 Nov 1;12(11):1612. doi: 10.3390/biom12111612 (PMC9687636; doi:10.3390/biom12111612)
Supplement: Supplementary file 1 [file biomolecules-12-01612-s001.zip › biomolecules-1987855-supplementary.pdf]

Supplementary File

Computational Investigation of 1, 3, 4 oxadiazole derivatives as Lead Inhibitors of VEGFR 2 in comparison with EGFR: Density Functional Theory, Molecular docking and Molecular Dynamic Simulation Studies

Muhammad Sajjad Bilal[1], Syeda Abida Ejaz[1]*, Seema Zargar[2] Naveed Akhtar[3], Tanveer A. Wani[4], Naheed Riaz[5], Adullahi Tunde Aborode[6], Farhan Siddique[7,8], Nojood, Altwaijry[2], Hammad M. Al-Kahtani[4] and Haruna Isiyaku Umar[9],[10]

1-Department of Pharmaceutical Chemistry, Faculty of Pharmacy, The Islamia University of Bahawalpur, Bahawalpur 63100, Pakistan
2-Department of Biochemistry, College of Science, King Saud University, P.O. Box 22452, Riyadh 11451, Sau-di Arabia
3-Department of Pharmaceutics, Faculty of Pharmacy, The Islamia University of Bahawalpur, Bahawalpur 63100, Pakistan
4-Department of Pharmaceutical Chemistry, College of Pharmacy, King Saud University, P.O. Box 2457, Ri-yadh 11451, Saudi Arabia
5-Department of Chemistry, Baghdad-ul-Jadeed Campus, The Islamia University of Bahawalpur, Bahawal-pur, Pakistan, 63100
6-Department of Chemistry, Mississippi State University, Starkville, USA
7-Laboratory of Organic Electronics, Department of Science and Technology, Linköping University, SE-60174 Norrköping, Sweden
8-Department of Pharmaceutical Chemistry, Faculty of Pharmacy, Bahahuddian Zakariya University, Multan, 60800, Pakistan
9-Molecular Biology and Bioinformatics Laboratory, Department of Biochemistry, Federal University of Technology, PMD 704, Akure, Ondo State, Nigeria
10-Computer-aided therapeutic discovery and design (CATDD)platform, Federal University of Technology, PMD 704, Akure, Ondo State, Nigeria
*Corresponding Authors:
Syeda Abida Ejaz; abida.ejaz@iub.edu.pk; abidaejaz2010@gmail.com

To whom correspondence should address:

Dr. Syeda Abida Ejaz; *Department of Pharmaceutical Chemistry, Faculty of Pharmacy, The Islamia University of Bahawalpur, Pakistan.*, Postal Code 63100, Pakistan Tel: +92-062-9250245 Fax: +92-062-9250245, E-Mail: abida.ejaz@iub.edu.pk; abidaejaz2010@gmail.com

Table S1: Chemical structures of oxadiazoles with IUPAC names.

	Solubility	Substituted 1,3,4-0xadiazoles-	Molecular Formula/ Molecular Mass	IUPAC NAME
**1**	CH_3OH	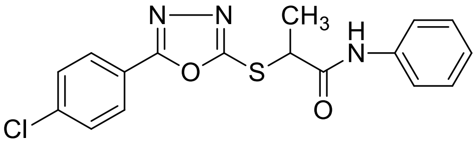	$C_{17}H_{14}ClN_3O_2S$/359.05	2-((5-(4-chlorophenyl)-1,3,4-oxadiazol-2-yl)thio)-N-phenylpropanamide
**2**	CH_3OH	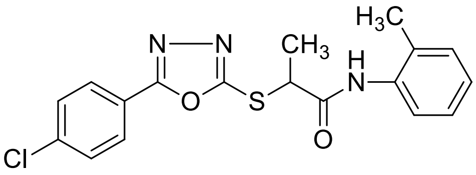	$C_{18}H_{16}ClN_3O_2S$/373.07	2-((5-(4-chlorophenyl)-1,3,4-oxadiazol-2-yl)thio)-N-(o-tolyl)propanamide
**3**	CH_3OH	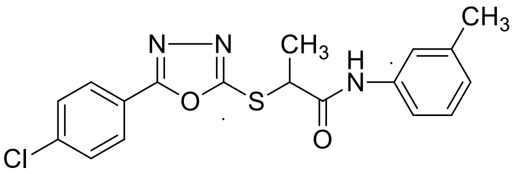	$C_{18}H_{16}ClN_3O_2S$/373.07	2-((5-(4-chlorophenyl)-1,3,4-oxadiazol-2-yl)thio)-N-(m-tolyl)propanamide
**4**	CH_3OH	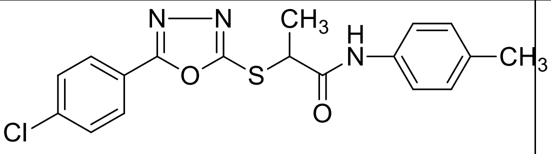	$C_{18}H_{16}ClN_3O_2S$/373.07	2-((5-(4-chlorophenyl)-1,3,4-oxadiazol-2-yl)thio)-N-(p-tolyl)propanamide
**5**	CH_3OH	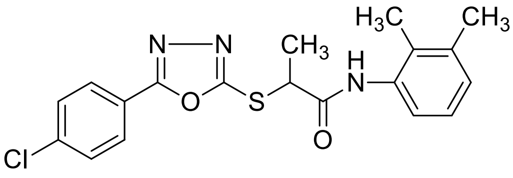	$C_{19}H_{18}ClN_3O_2S$/387.08	2-((5-(4-chlorophenyl)-1,3,4-oxadiazol-2-yl)thio)-N-(2,3-dimethylphenyl)propanamide
**6**	CH_3OH	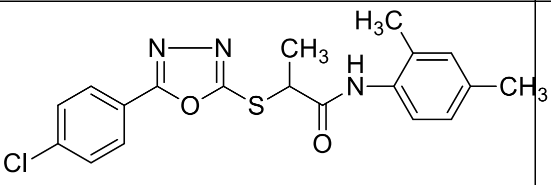	$C_{19}H_{18}ClN_3O_2S$/387.08	2-((5-(4-chlorophenyl)-1,3,4-oxadiazol-2-yl)thio)-N-(2,4-dimethylphenyl)propanamide

**7**	CH_3OH	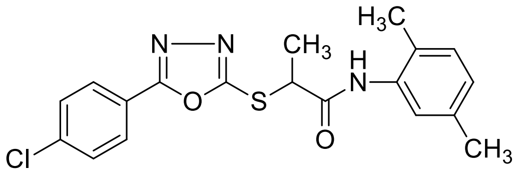	$C_{19}H_{18}ClN_3O_2$ S/387.08	2-((5-(4-chlorophenyl)-1,3,4-oxadiazol-2-yl)thio)-N-(2,5-dimethylphenyl)propanamide
**8**	CH_3OH	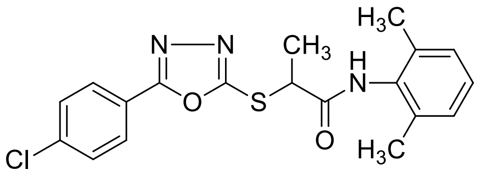	$C_{19}H_{18}ClN_3O_2$ S/387.08	2-((5-(4-chlorophenyl)-1,3,4-oxadiazol-2-yl)thio)-N-(2,6-dimethylphenyl)propanamide
**9**	CH_3OH	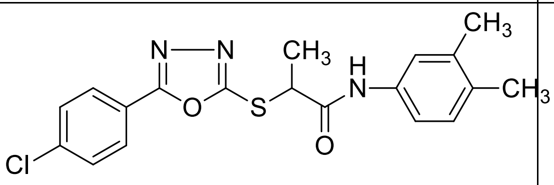	$C_{19}H_{18}ClN_3O_2$ S/387.08	2-((5-(4-chlorophenyl)-1,3,4-oxadiazol-2-yl)thio)-N-(3,4-dimethylphenyl)propanamide
**10**	CH_3OH	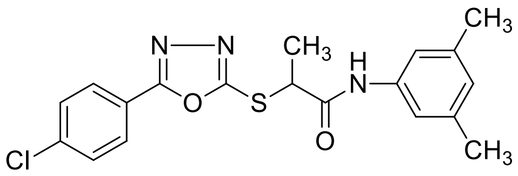	$C_{19}H_{18}ClN_3O_2$ S/387.08	2-((5-(4-chlorophenyl)-1,3,4-oxadiazol-2-yl)thio)-N-(3,5-dimethylphenyl)propanamide
**11**	CH_3OH	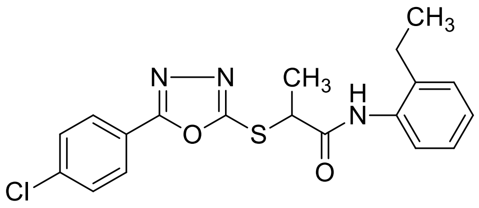	$C_{19}H_{18}ClN_3O_2$ S/387.08	2-((5-(4-chlorophenyl)-1,3,4-oxadiazol-2-yl)thio)-N-(2-ethylphenyl)propanamide
**12**	CH_3OH	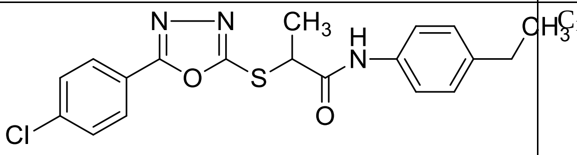	$C_{19}H_{18}ClN_3O_2$ S/387.08	2-((5-(4-chlorophenyl)-1,3,4-oxadiazol-2-yl)thio)-N-(4-ethylphenyl)propanamide
**13**	CH_3OH	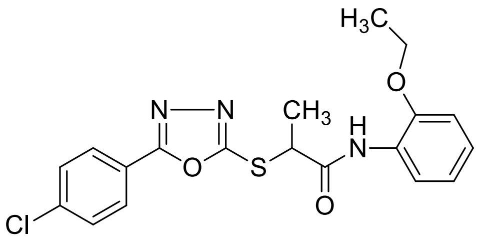	$C_{19}H_{18}ClN_3O_3$ S/403.08	2-((5-(4-chlorophenyl)-1,3,4-oxadiazol-2-yl)thio)-N-(2-ethoxyphenyl)propanamide
**14**	CH_3OH	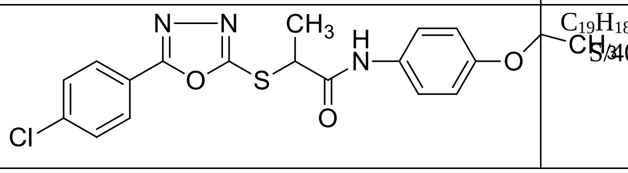	$C_{19}H_{18}ClN_3O_3$ S/403.08	2-((5-(4-chlorophenyl)-1,3,4-oxadiazol-2-yl)thio)-N-(4-ethoxyphenyl)propanamide

**Vegfr ligand**		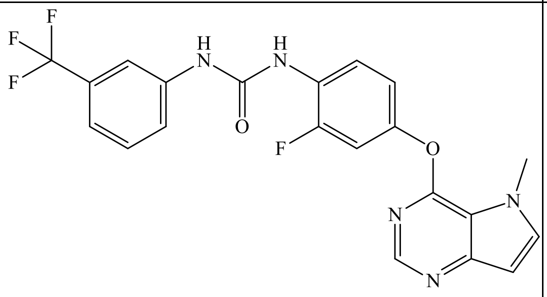		1-{2-fluoro-4-[(5-methyl-5H-pyrrolo[3,2-d]pyrimidin-4-yl)oxy]phenyl}-3-[3-(trifluoromethyl)phenyl]urea

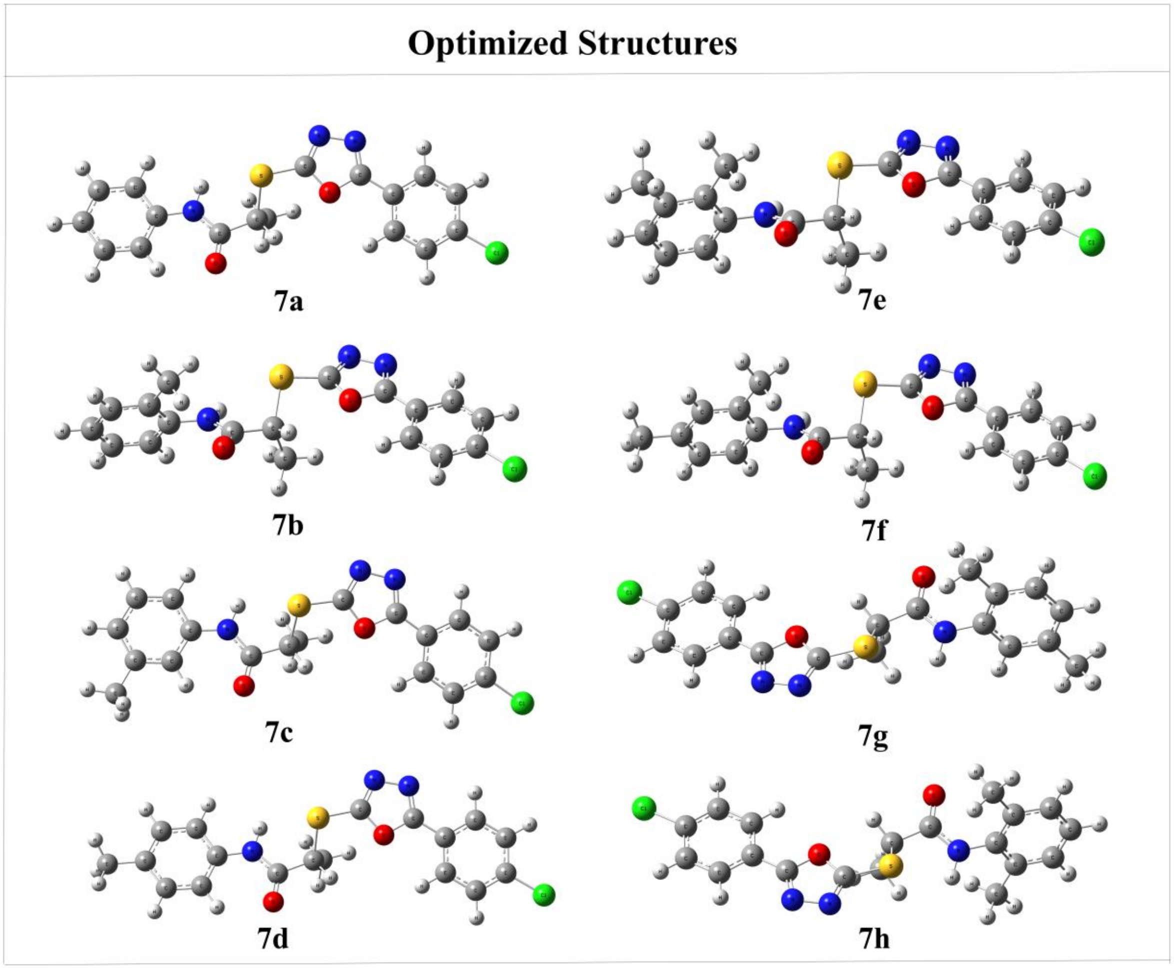

Figure S1: Optimized structures of the oxadiazoles derivatives **(7a-7h)** in gas phase.

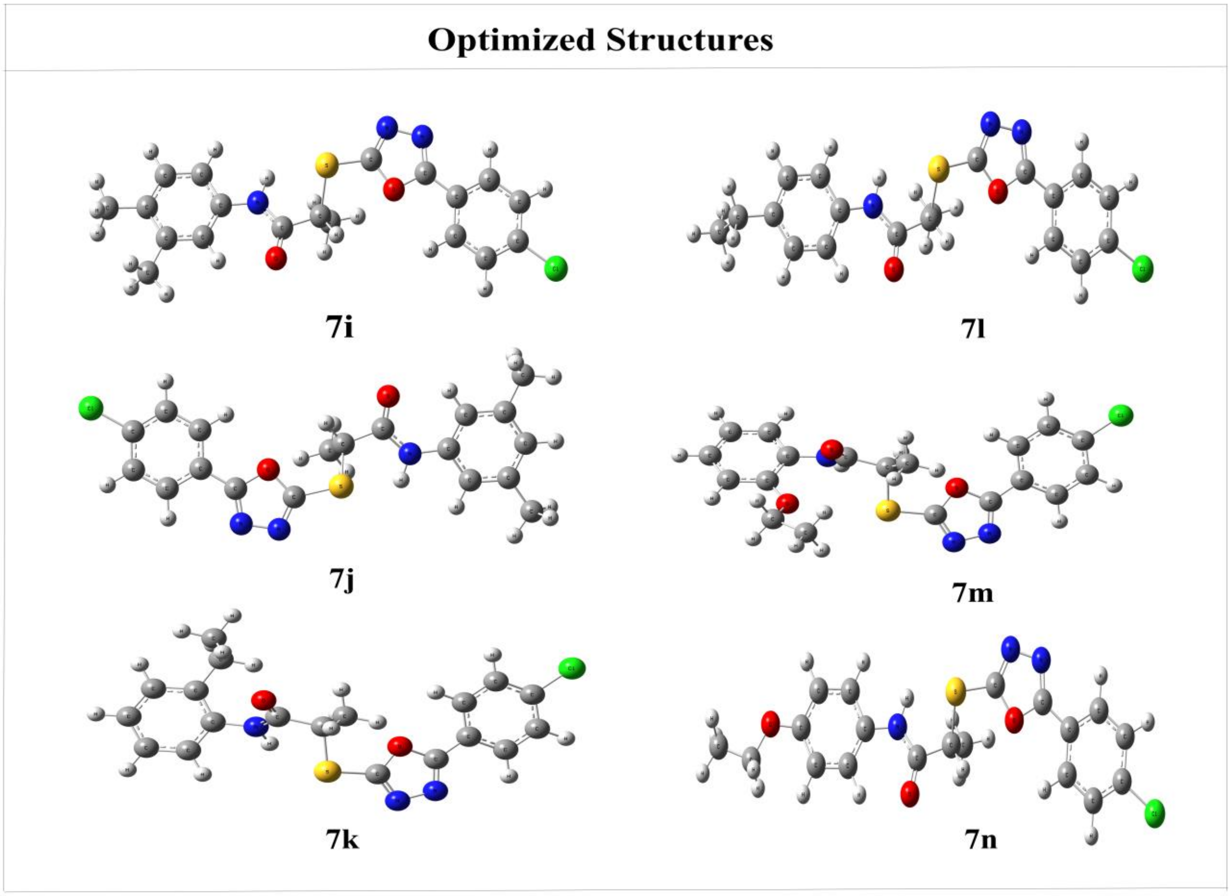

Figure S2: Optimized structures of the oxadiazoles derivatives **(7i-7n)** in gas phase.

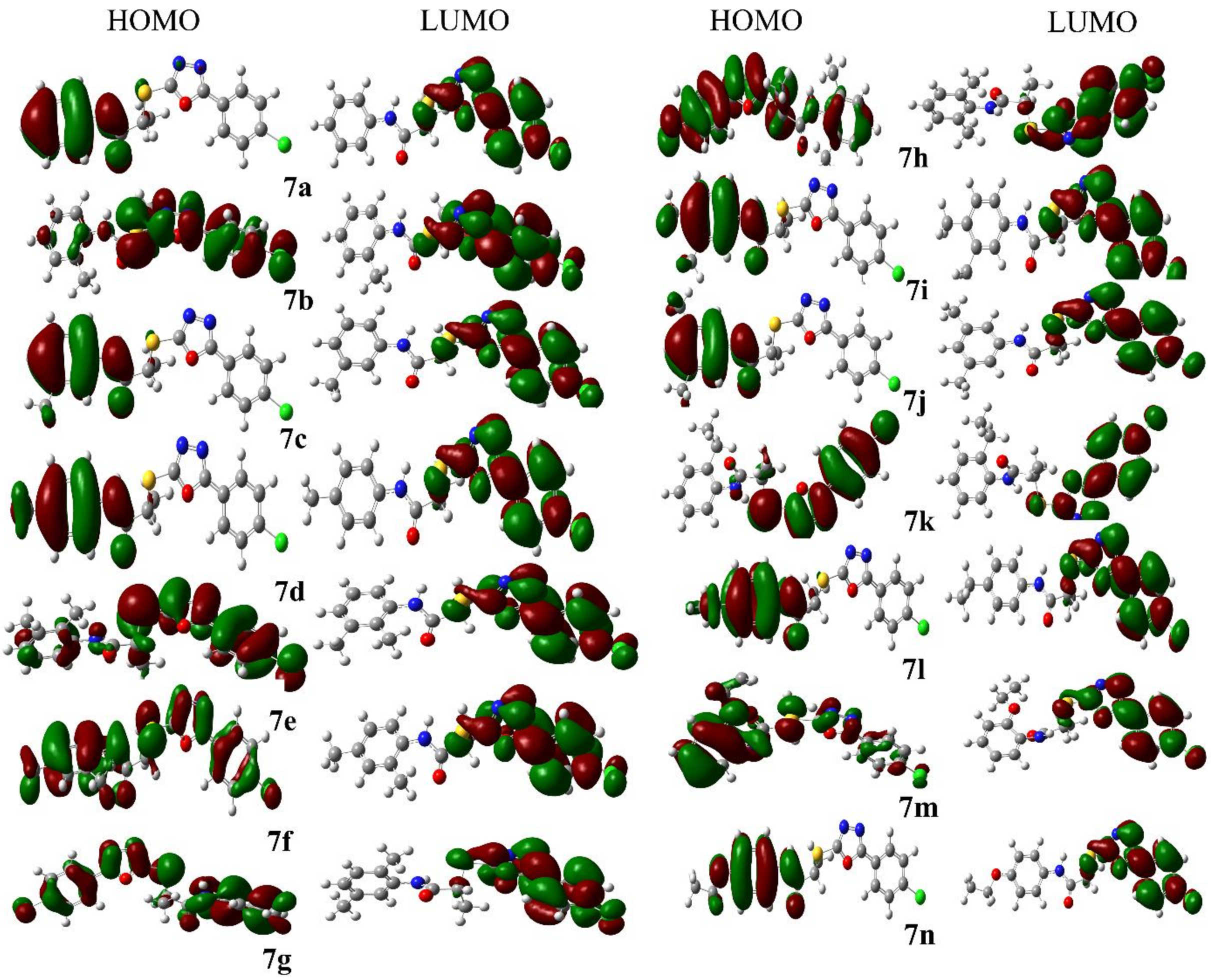

Figure S3: HOMO and LUMO structures of all the oxadiazoles derivatives in gas phase.

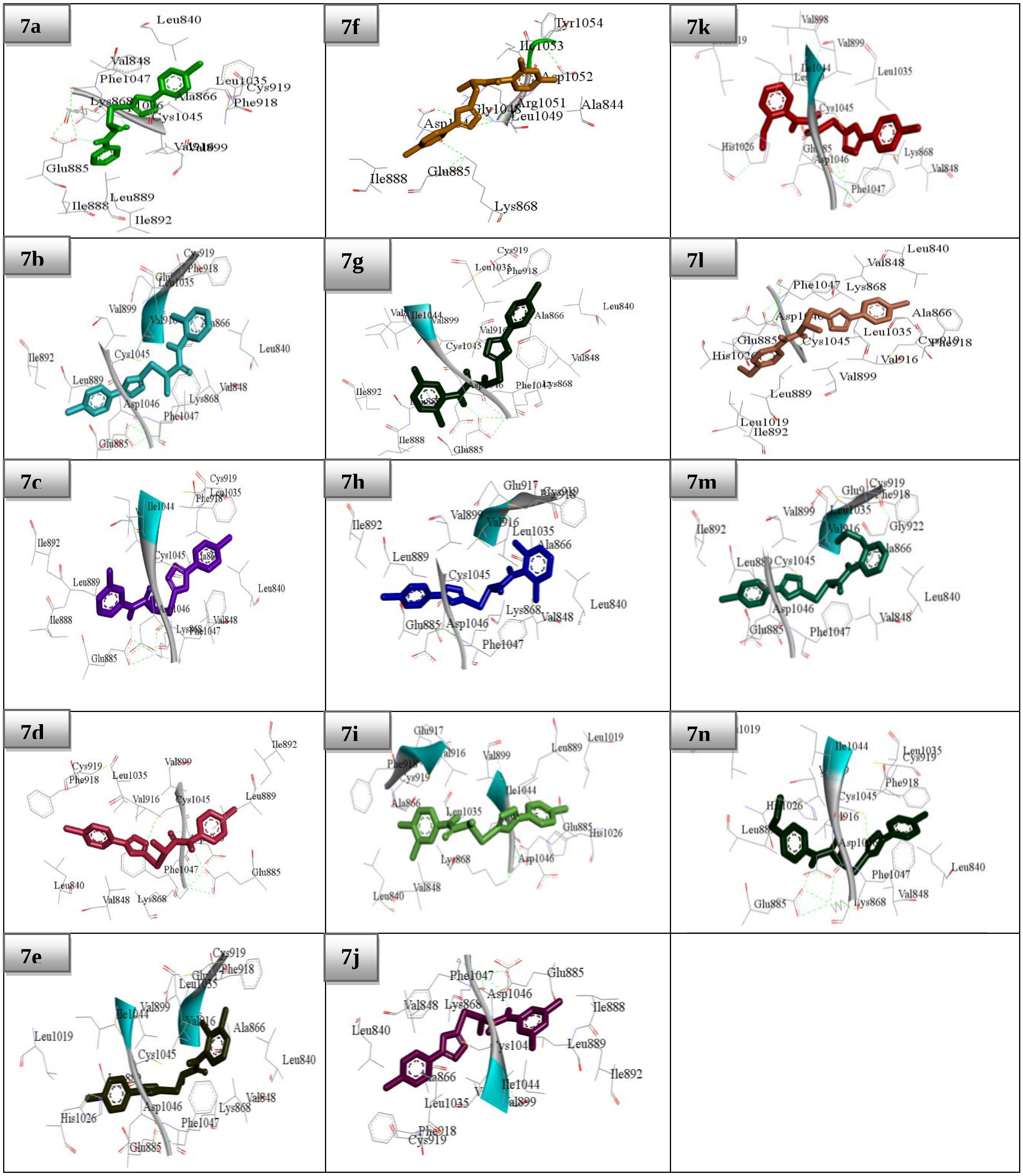

Figure S4: 3D binding interactions of all compounds with VEGFR

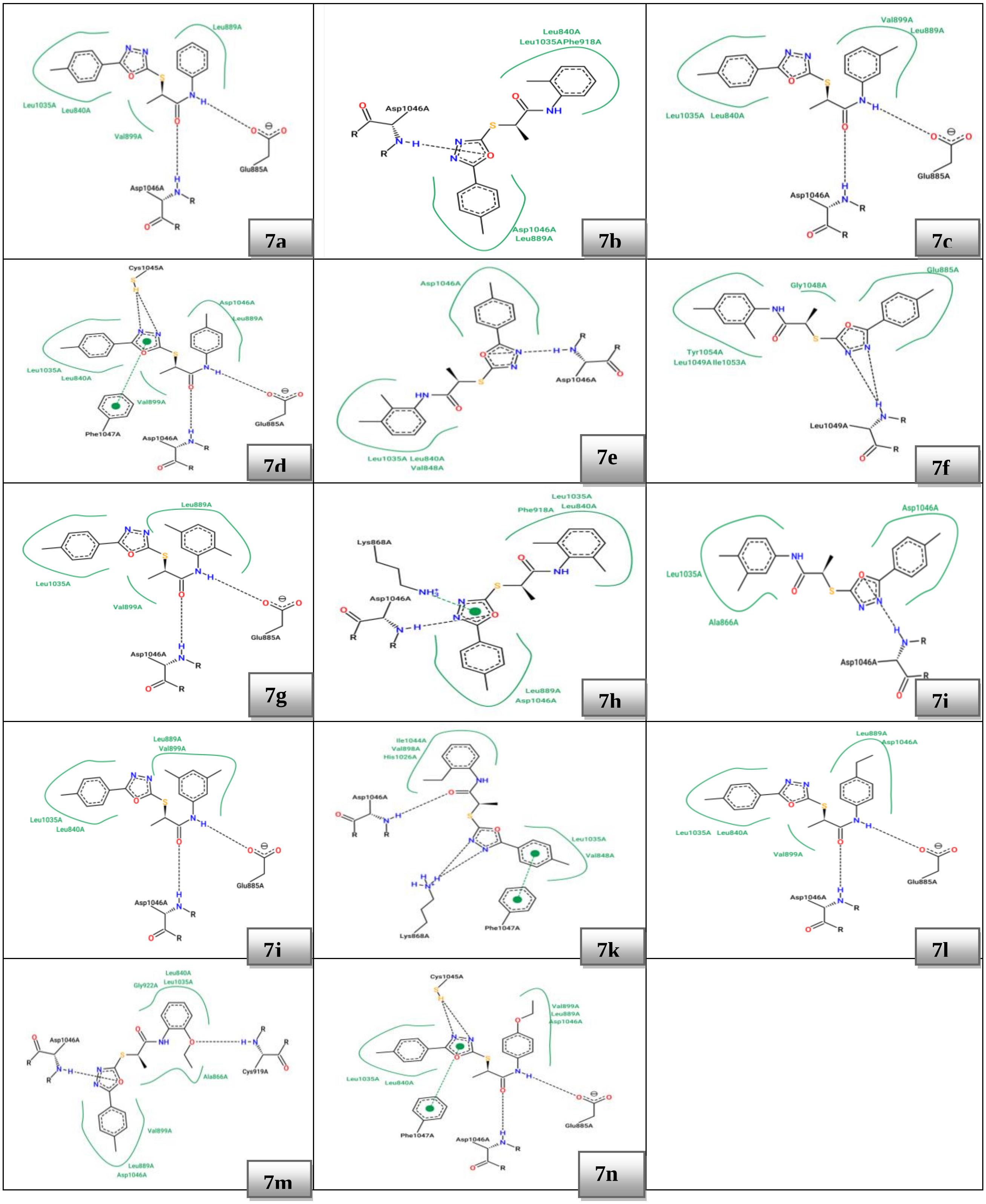

Figure S5: 2D binding interactions of all compounds with VEGFR

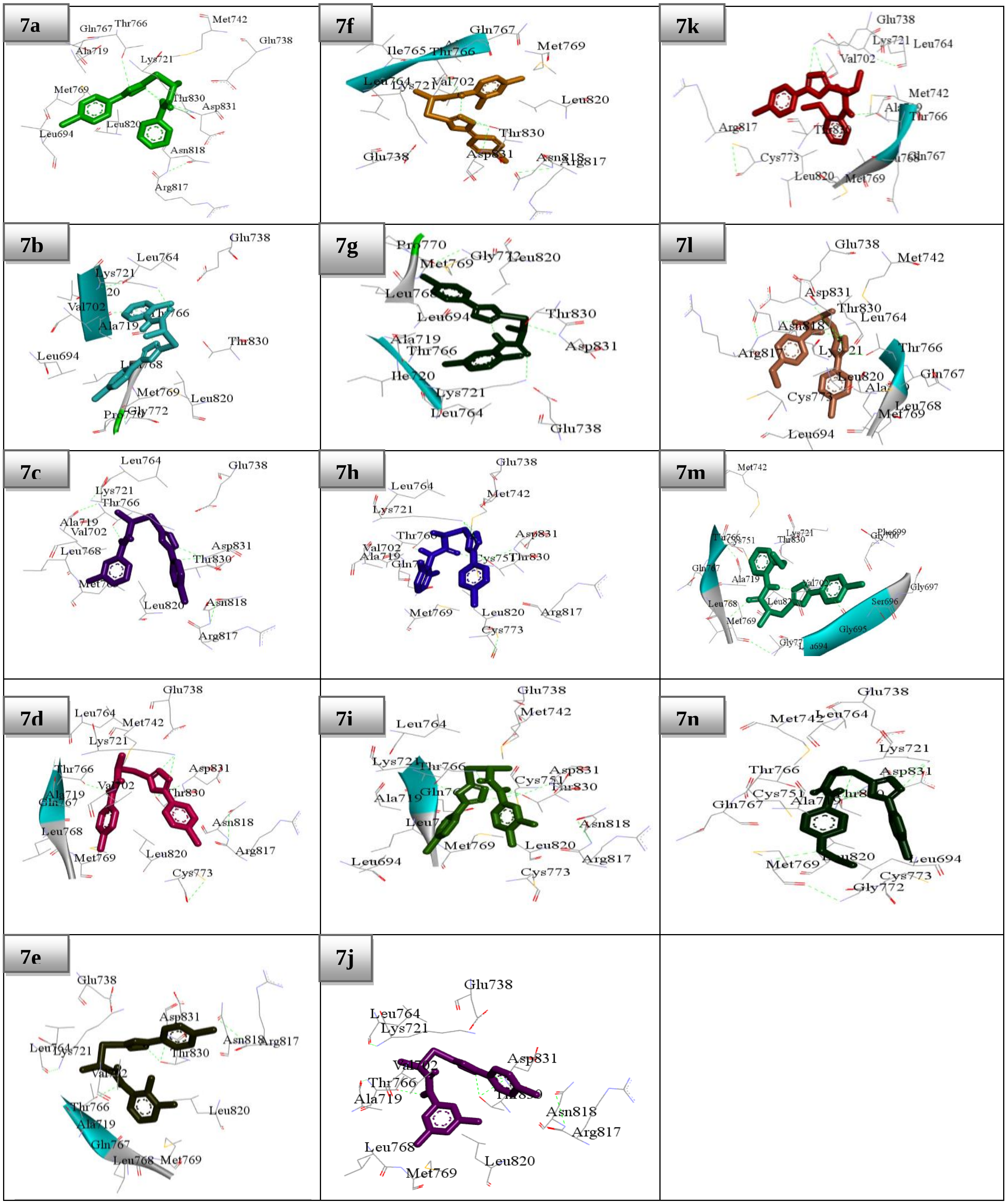

Figure S6: 3D binding interactions of all compounds with EGFR

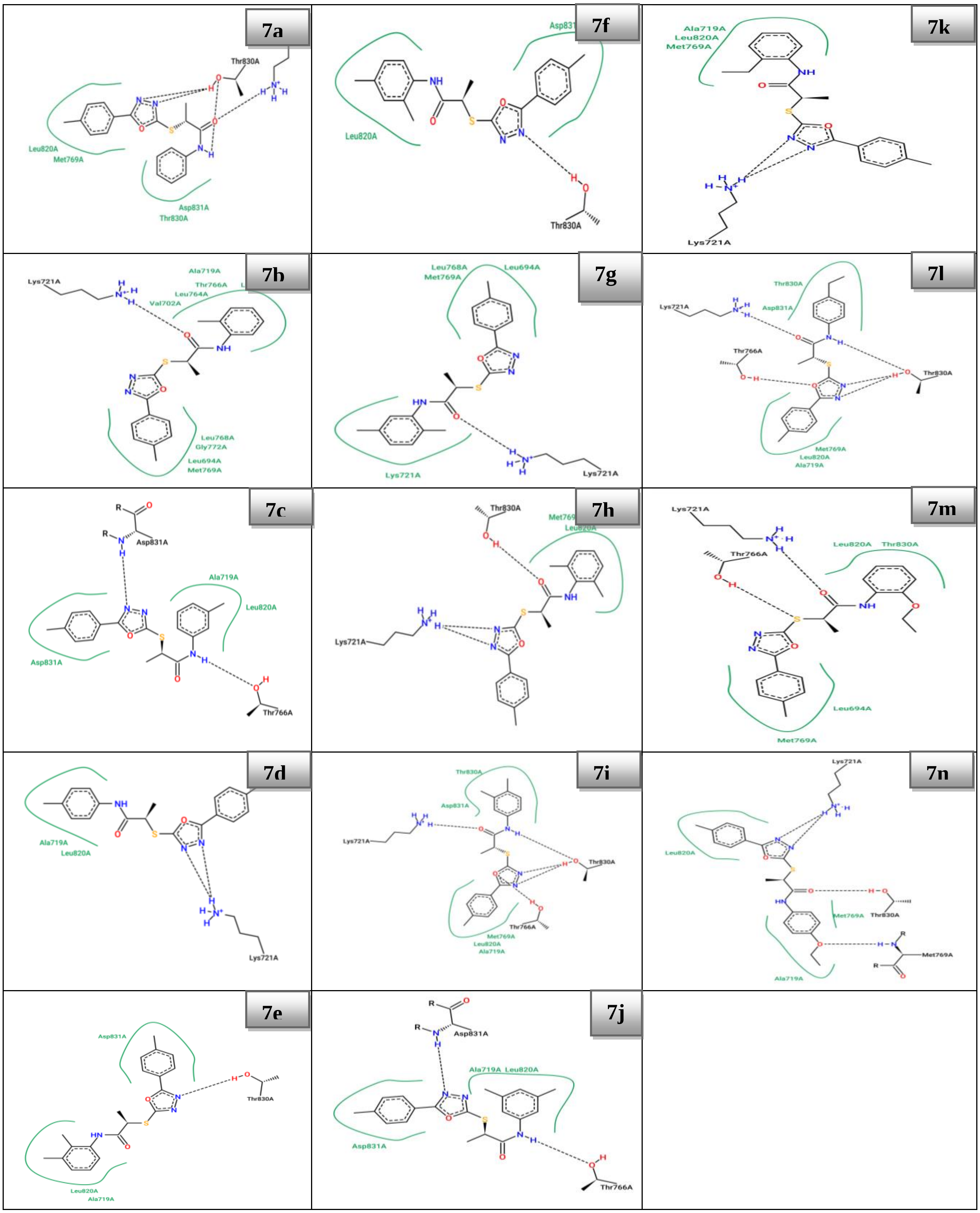

Figure S7: 2D binding interactions of all compounds with EGFR

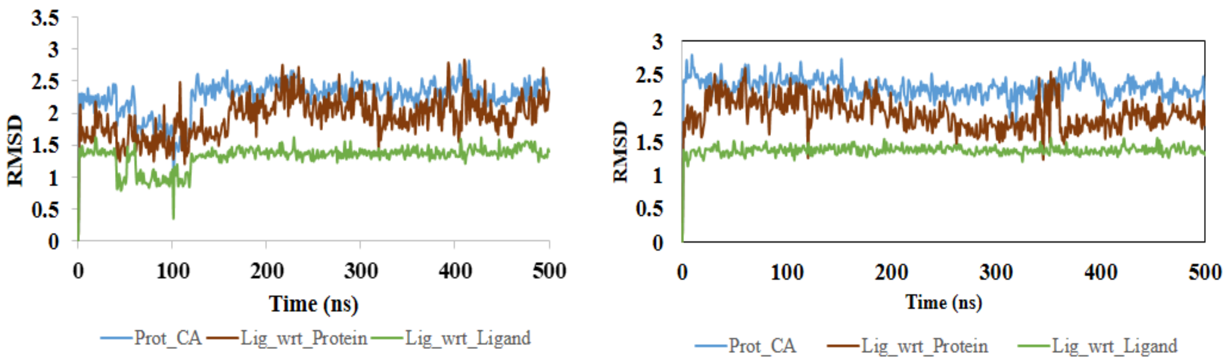

A B

Figure S8: Simulation replicate of Vegfr-7j Complex

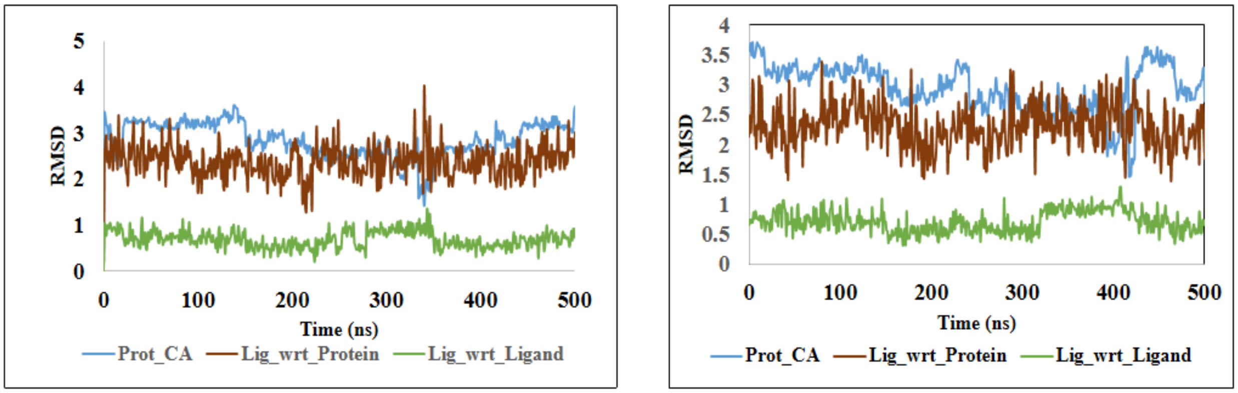

A B

Figure S9: Simulation replicate of Egfr-7j Complex
